# Characterization of raloxifene as a potential pharmacological agent against SARS-CoV-2 and its variants

**DOI:** 10.1038/s41419-022-04961-z

**Published:** 2022-05-25

**Authors:** Daniela Iaconis, Licia Bordi, Giulia Matusali, Carmine Talarico, Candida Manelfi, Maria Candida Cesta, Mara Zippoli, Francesca Caccuri, Antonella Bugatti, Alberto Zani, Federica Filippini, Laura Scorzolini, Marco Gobbi, Marten Beeg, Arianna Piotti, Monica Montopoli, Veronica Cocetta, Silvia Bressan, Enrico M. Bucci, Arnaldo Caruso, Emanuele Nicastri, Marcello Allegretti, Andrea R. Beccari

**Affiliations:** 1 Dompé farmaceutici S.p.A., Naples, Italy; 2 National Institute for Infectious Diseases Lazzaro Spallanzani-IRCCS, Rome, Italy; 3 Dompé farmaceutici S.p.A., L’Aquila, Italy; 4 Department of Molecular and Translational Medicine, Section of Microbiology and Virology, University of Brescia Medical School, Brescia, Italy; 5 Department of Biochemistry and Molecular Pharmacology, Istituto di Ricerche Farmacologiche Mario Negri IRCCS, Milan, Italy; 6 Department of Pharmaceutical and Pharmacological Sciences, University of Padua, Padua, VIMM Veneto Institute Molecular Medicine, Padua, Italy; 7 Sbarro Health Research Organization, Biology Department CFT, Temple University, Philadelphia, PA USA

**Keywords:** Viral infection, Preclinical research

## Abstract

The new coronavirus SARS-CoV-2 is the causative agent of the COVID-19 pandemic, which so far has caused over 6 million deaths in 2 years, despite new vaccines and antiviral medications. Drug repurposing, an approach for the potential application of existing pharmaceutical products to new therapeutic indications, could be an effective strategy to obtain quick answers to medical emergencies. Following a virtual screening campaign on the most relevant viral proteins, we identified the drug raloxifene, a known Selective Estrogen Receptor Modulator (SERM), as a new potential agent to treat mild-to-moderate COVID-19 patients. In this paper we report a comprehensive pharmacological characterization of raloxifene in relevant in vitro models of COVID-19, specifically in Vero E6 and Calu-3 cell lines infected with SARS-CoV-2. A large panel of the most common SARS-CoV-2 variants isolated in Europe, United Kingdom, Brazil, South Africa and India was tested to demonstrate the drug’s ability in contrasting the viral cytopathic effect (CPE). Literature data support a beneficial effect by raloxifene against the viral infection due to its ability to interact with viral proteins and activate protective estrogen receptor-mediated mechanisms in the host cells. Mechanistic studies here reported confirm the significant affinity of raloxifene for the Spike protein, as predicted by in silico studies, and show that the drug treatment does not directly affect Spike/ACE2 interaction or viral internalization in infected cell lines. Interestingly, raloxifene can counteract Spike-mediated ADAM17 activation in human pulmonary cells, thus providing new insights on its mechanism of action. A clinical study in mild to moderate COVID-19 patients (NCT05172050) has been recently completed. Our contribution to evaluate raloxifene results on SARS-CoV-2 variants, and the interpretation of the mechanisms of action will be key elements to better understand the trial results, and to design new clinical studies aiming to evaluate the potential development of raloxifene in this indication.

## Introduction

Coronaviruses are the causative agents of multiple respiratory and intestinal infection in humans and animals [1, 2], and SARS-CoV-2 is able to cause severe acute respiratory illness, multi-organ failure up to the death [3, 4]. In addition, prolonged prothrombin times, mild thrombocytopenia and elevated D-dimer values were observed together with lymphocytopenia, also suggested as a predictor of prognosis [5, 6].

As of March 24^th,^ 2022, SARS-CoV-2 infection led to more than 6 million deaths worldwide (https://covid19.who.int/) and 7.5% of COVID-19 cases reported by EU/EEA countries to the European Surveillance System (TESSy) were hospitalized.

The current COVID-19 pandemic is believed to have initially developed in an animal host: SARS-CoV-2 shares high genome similarity with betacoronaviruses isolated from a bat (RaTG13) and from a Malayan pangolin. Therefore, SARS-CoV-2 has been hypothesized to have originated in bats and gone through multiple recombination events while migrating through other mammals [7]. It is increasingly evident that animals are an important epidemiological part of this pandemic in transmission and appearance of viral variants [8, 9] well as in the new mechanisms of infection that gradually emerge [10]. To date, notwithstanding the advent of antivirals, vaccine programs [11] and social distancing interventions, it is believed that the virus will most likely become endemic [12]. In addition, the emerging of SARS-CoV-2 variants raises great concern for vaccine efficacy, reinfection events, increased transmissibility and disease severity. As the virus started to spread, a mutated Spike SARS-CoV-2 variant (D614G) emerged which was associated with increased infectivity, becoming the predominant variant in Europe and worldwide without any increase in disease severity [13, 14]. During the last year, other variants were defined as “variants of concern” (VOC). Some of them are considered of high clinical relevance like B.1.1.7 (UK, alpha), B.1.351 (South African, beta), B.1.1.28 (Brazilian P.1, gamma), B.1.427 and B.1.429 (Californian, epsilon), characterized by increased transmissibility, immune evasion and higher virulence [15–18]. In May 2021, the B.1.617.2 (Indian, Delta) was added to the WHO list of VOCs and described as more transmissible (up to 50% than the alpha variant), able to escape adaptive immunity, and to cause sharp rises in infections in many countries, including those with relatively high vaccination coverage [19, 20]. As of November 26th 2021, the newly emerged B.1.1.529 variant, firstly identified in South-Africa, was classified as “omicron” VOC (https://www.who.int/news/item/28-11-2021-update-on-omicron), characterized by higher transmissibility than other VOCs but milder symptoms, mainly of the upper respiratory tract [21]. The growing relevance of new VOCs stimulated further investigations and new impetus to develop broad-spectrum drugs or vaccines for long-term prevention, treatment and control of COVID-19. The virus entry machinery has been considered a privileged target to identify potential therapeutic targets and in this context several preclinical studies and clinical trials are ongoing [22]. Recent evidence shows that Nuclear Receptors (NRs), and in particular sex hormone receptors like estrogen (ER) and androgen (AR) receptors, could be involved in determining the outcome of COVID-19 infection because able to regulate the viral entry protein expression and activity [23–25]. A protective effect of estrogens in the progression of COVID-19 infection has been associated with their role in the regulation of innate and adaptive immune responses, as well as in the control of the cytokine storm [26–32], whereas the activation of androgen receptors seems to correlate with the worse COVID-19 clinical outcome observed in men [25, 33–35]. Recently, in the context of the H2020 project EXSCALATE4CoV (E4C), an extensive virtual screening campaign on SARS-CoV-2 target proteins based on the EXSCALATE platform, a tool for drug repurposing [36–38], allowed to identify several molecules active against SARS-CoV-2. Raloxifene, a well-known SERM [39–42], was selected following this approach. Besides a direct inhibition of viral protein functions, several papers propose the use of raloxifene and other SERMs as potential therapeutic strategy to treat paucisymptomatic COVID-19 due to the multiple links between ER modulation and host response to viral infections [43] that suggest beneficial effects both in controlling viral replication in early post-infection phase, and in preventing/attenuating the cytokine storm and inflammation in more severe COVID-19 [44]. Further, a large body of literature points to a protective role of the ER-signaling as relevant in the observed gender sensitivity, as additional boost to develop SERMs, specifically raloxifene, in this indication [43]. Raloxifene is a drug registered for the treatment and prevention of osteoporosis and risk of invasive breast cancer in postmenopausal women [45], ER agonist in the bone, liver and cardiovascular system, and ER antagonist in reproductive tissues [46]. This tissue specificity allows for its use in postmenopausal osteoporosis and prevention of breast cancer without increasing the risk of endometrial cancer [47, 48]. Raloxifene was also studied in men for treatment of diseases as schizophrenia, prostate cancer and osteoporosis [49, 50]. Interestingly, raloxifene effect was characterized against Ebola [51, 52], Hepatitis C [53, 54], Hepatitis B [55], Zika [56], Influenza A viruses [57], and as adjuvant antiviral treatment of chronic Hepatitis C in women [58]. Raloxifene in vitro activity on SARS-CoV-2 was previously reported [59], and the drug was observed to reduce infectivity in a dose-dependent manner (IC_50_ value of 7.1 μM). Here we report a full characterization of the antiviral activity of raloxifene on SARS-CoV-2 in two cellular contexts, Vero E6 and Calu-3 cell lines, testing the efficacy of the treatment on all the most relevant VOCs, and clearly showing the independence of raloxifene activity from viral variant specificity. Furthermore, mechanistic studies shed light on its mechanism of action supporting the concept that a pleiotropic mechanism may account for its inhibition of viral replication. In particular, the ability of raloxifene to modulate the Spike-induced ADAM17 expression may account for a potential protection of pulmonary damage in consideration of the key role of ADAM-17 in virus entry and replication, and pathophysiological consequences in the lungs due to its excessive activation in SARS-CoV-2 infection [60].

Taken together, this evidence supports the concept that raloxifene may exert a protective effect on SARS-CoV-2 infected patients through a pleiotropic mechanism of action. A Phase 2/3 clinical trial (NCT05172050) in outpatients with mild to moderate COVID-19 has been recently completed (Nicastri E. and et al., 2022 eClinicalMedicine; accepted for publication), and the results will hopefully give additional insights on the therapeutic potential of raloxifene.

## Materials and methods

All information is reported in Supplementary Information.

## Results

### In vitro effects of raloxifene on metabolism and SARS-CoV-2 infection in different cell lines

**Fig. 1:**
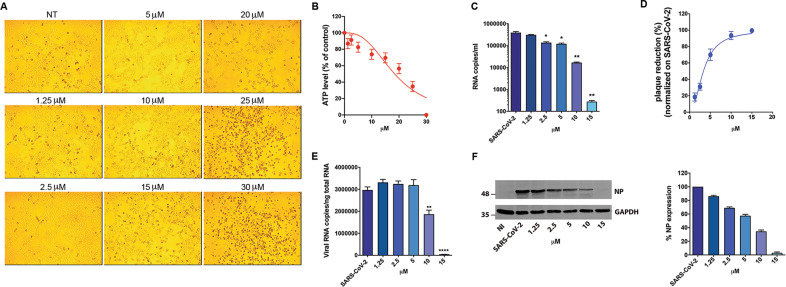
Effect of raloxifene on Vero E6 cells. Vero E6 cells were cultured for 48 h in the absence or in the presence of raloxifene at different concentrations. **A** 10× bright‐field images of Vero E6 cells after incubation for 48 h at 37 °C with the indicated raloxifene concentrations. **B** CellTiter-Glo was used to measure the antimetabolic effect of raloxifene. **C-F** Vero E6 cells were infected with SARS-CoV-2 and cultured in the absence or in the presence of different doses of raloxifene. **C** Viral yield in cell supernatants was quantitated by qRT-PCR. **D** Viral titer in cell supernatants was evaluated by plaque assay and plotted as percentage of plaque reduction compared to SARS-CoV-2. **E** Quantitation of SARS-CoV-2 genomes at the intracellular level by qRT-PCR. **F** NP expression in infected cells was analyzed by western blot (left panel). Densitometric analysis of western blot is shown in the right panel. Graph represents the percentage of NP expression. All the experiments were performed at least in three independent replicates and pictures shown are representative. Data are presented as the mean ± standard error of the mean *P < 0.05; **P < 0.01; ****P < 0.0001.

Vero E6 cells were cultured for 48 h in the absence or presence of different raloxifene concentrations. Raloxifene-treated cells showed a normal surface-adherent phenotype until the concentration of 15μM (Fig. 1A). A drug-dependent cytopathic effect (CPE) was evident at 20μM, involving the entire monolayer at 25μM and 30μM. At the same time, raloxifene showed a slight effect on the extent of cellular ATP accumulation at a concentration of 1.25 μM to 15 μM (87% and 70%, respectively). At higher doses, raloxifene showed a dose-dependent effect on ATP accumulation, reaching 56%, 35% and 0.6% at 20 µM, 25 µM and 30 µM, respectively (Fig. 1B). The half-maximal cytotoxic concentration (CC_50_) in Vero E6 cells was determined to be 18.4 µM.

Further, an apoptosis assay by flow cytometry was conducted at different doses of raloxifene (1.25, 2.5, 5, 10, 15, 20, 25 and 30 µM) in VERO E6 cells. As shown in Fig. S3 (Supplementary Information), raloxifene did not induce apoptosis at concentrations ranging from 1.25 to 20 μM, while it triggered a significant induction of apoptosis at 25 and 30 μM (36% and 45%, respectively).

Preliminary experiments were conducted to evaluate the effect of raloxifene in the viral pre-entry, entry or post-entry phases. Raloxifene displayed antiviral activity in the post-entry phase only (Fig. S1). Next, Vero E6 cells were infected with SARS-CoV-2 (B.1 lineage) at a multiplicity of infection (MOI) of 0.05, and 1 h p.i. (postinfection) cultured in the absence or presence of different drug concentrations. Raloxifene efficiently inhibited viral replication. Viral genome copy numbers evaluated by qRT-PCR at 48 h p.i., showed a significant reduction of the virus yield already at a concentration of 2.5μM (2.9-fold reduction), with a maximal reduction at 15 µM (1400-fold reduction) (Fig. 1C). Raloxifene also displayed a dose-dependent inhibition of viral replication, as determined by infectious viral titers, exhibiting a 70% reduction of viral titer at a concentration of 5 µM, with 94% to 100% inhibition at 10 µM and 15 µM, respectively (Fig. 1D). Its efficacy was then confirmed at intracellular level; quantification of viral RNA in SARS-CoV-2-infected cells showed a significant reduction of intracellular viral genome copy number at 10 µM, and a 99-fold reduction at 15 µM (Fig. 1E). Accordingly, western blot (WB) analysis showed a viral dose-dependent inhibition upon raloxifene treatment with 43% reduction of NP viral protein expression at a concentration of 5 µM, with 65% and 97% reduction at 10 µM and 15 µM, respectively (Fig. 1F and S2). The half-maximal inhibitory concentration (IC_50_) was calculated to be 3.3 µM, while the selectivity index (SI) was calculated to be 5.6.

**Fig. 2:**
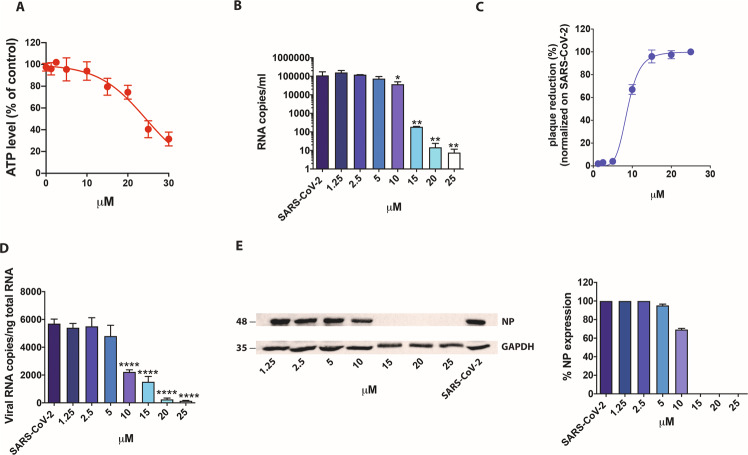
Effect of raloxifene on Calu-3 cells. Calu-3 cells were cultured for 48 h in absence or in the presence of raloxifene at different concentrations. **A** CellTiter-Glo was used to measure the antimetabolic effect of raloxifene. **B**–**E** Cells were infected with SARS-CoV-2 and cultured in the absence or in the presence of different doses of raloxifene. **B** Viral yield in cell supernatants was quantitated by qRT-PCR. **C** Viral titer in cell supernatants was evaluated by plaque assay and plotted as percentage of plaque reduction compared to SARS-CoV-2. **D** Quantitation of SARS-CoV-2 genomes at the intracellular level by qRT-PCR. **E** Nucleocapsid (NP) protein expression in infected cells was analyzed by western blot (left panel). Densitometric analyses of western blot results are shown. Graph represents the percentage of NP protein expression. All the experiments were performed at least in three independent replicates and pictures shown are representative. Data are presented as the mean + standard error of the mean *P < 0.05; **P < 0.01; ****P < 0.0001.

We then tested raloxifene on Calu-3 cells. The CC_50_ value was found to be 24.4 µM (Fig. 2A). Next, Calu-3 cells were infected as described above, and supernatants collected at 48 h p.i. and tested by qRT-PCR. The treatment significantly reduced the virus yield (Fig. 2B). Raloxifene displayed a dose-dependent inhibition of viral replication, as determined by infectious viral titers, exhibiting a 67% reduction of viral titer at a concentration as low as 10 µM, with 96% and 98% inhibition at drug concentrations of 15 µM and 25 µM, respectively. The efficacy of the treatment was confirmed at intracellular level by qRT-PCR and WB on NP (Fig. 2C-E and S3). The IC_50_ was calculated and found to be 9 µM, while SI was calculated to be 2.7.

### Raloxifene exerts antiviral activity on SARS-CoV-2 variants

**Fig. 3:**
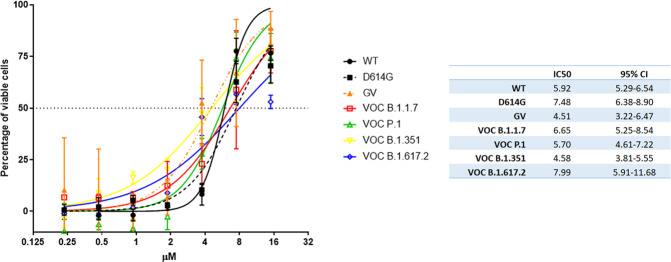
Raloxifene reduces the CPE induced by SARS-CoV-2 variants in Vero E6 cells. The graph shows the inhibition of CPE observed at different concentration of raloxifene on different VOCs. The IC_50_ values calculated by nonlinear regression are shown in the table. Percentage of viable cells calculated on not treated not infected = 100%; not treated SARS-CoV-2 infected cells= 0%. Bars indicate SD.

We then performed a systematic study of the antiviral efficacy of raloxifene on the most common variants in Vero E6 cells (Fig. 3). Different viral strains were used: the wild type (WT) isolated in January 2020 from Chinese patient, two different isolates for the D614G spike variants (named GV and D614G), and the main VOCs isolated in UK, Brazil, South Africa and India (VOC B1.1.7/alpha, VOC P.1/gamma, VOC B1.351/beta, and VOC B1.617.2/delta, respectively). We first determined the time window for each variant in which CPE appeared. It was evident at 48 h for all the tested strains but VOC B1.1.7 and VOC P.1, for which evident CPE appeared later (56 h and 72 h, respectively). In parallel, uninfected cells were cultured in presence of different doses of raloxifene to evaluate possible treatment-related cytotoxicity. In cells treated with 15μM drug, we observed a reduced percentage of viable cells (82.9 + /−8.69% and 76.3 + /−5.84%, at 48 h and 72 h respectively, compared to 100% in untreated cells). To determine antiviral efficacy of raloxifene, CPE was measured in infected cells treated with serial dilutions of raloxifene (15 to 0.23 μM) using the time windows identified for each strain. The drug was able to recover cell viability in Vero E6 cells infected with all the tested viral strains. The IC_50_ calculated on recovering of cell viability ranged from 4.50 to 7.99 μM depending on the strain (Fig. 3) with strong antiviral activity against all the tested variants.

### System Biology screening to investigate polypharmacological effects of raloxifene against SARS-CoV-2 infection

Aiming to gain insights on relevant pathways potentially modulated by raloxifene in the context of viral infection we built a molecular network connecting the human-virus interactome and proteins involved in COVID-19 pathogenesis (SI section). The resulting network was used to generate a list of proteins used as a probe for screening all papers on raloxifene in which proteins relevant for SARS-CoV-2 infection are cited. Three functional groups of human genes involved in the biology of viral infection that could be modulated by raloxifene were identified (Figure S2):

1. Genes connected to inflammation, including those modulated by the raloxifene molecular target ER2;
2. Genes expressed in the lungs which are modulated by ER receptors whose downregulation results associated with severe asthma, and whose unfavorable variants cause worse respiratory consequences, according to GWAS studies;
3. Genes directly modulated by the virus, both during the cell entry and the replication phase, including proteins upstream or downstream some of raloxifene-controlled pathways.

Within the first group of genes the cytokine Interleukin-6 (IL-6) [61] was identified, one of the clinically validated targets of the anti COVID-19 therapy. A raloxifene-mediated downregulation of IL-6 inflammatory signal and expression was found in a clinical setting [62]. Other serum cytokines (i.e. TNF-α and TGF-β1) involved in SARS-CoV-2 cytokine storm are also regulated by raloxifene.

The second group includes genes that regulate the production of nitric oxide involved in the vascular and respiratory response to the viral infection. Raloxifene can upregulate the expression of eNOS (NOS3) in rat thoracic aorta, and it is expected to exert a potentially important vasculo-protective effect, and to contribute to clinical improvements in ARDS and pulmonary hypertension [63], concept already tested in clinical trials also with other compounds [64].

As for the third group, a direct antiviral action of raloxifene in inhibiting viral replication and/or infection was found in different contexts like Ebola Virus [51, 52, 65], HBV [56], HCV [53], Influenza Virus A [57], and also in a clinical trial against HCV [58]. We found a group of genes directly modulated by the virus, both during the cell entry phase and the replication phase, which also include raloxifene-controlled pathways, ACE2, TMPRSS2 and ADAM17 [66]. ER mRNA levels are associated with these gene transcripts in the atrium, suggesting a role for ER in modulating the expression of proteins relevant for viral entry and expression.

### Virtual screening on SARS-CoV-2 proteins and SPR analysis: raloxifene among the best-scored binders

**Fig. 4:**
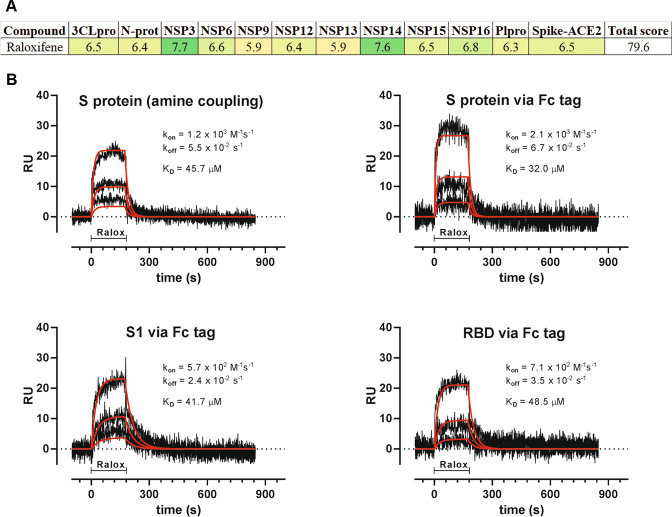
Raloxifene binds SARS-CoV-2 viral proteins. **A** LiGen™ docking score values that predict the binding affinity of the molecules in the protein binding site are reported in shades of green: dark green corresponds to higher values. Scores are also reported (the higher, the better). **B** SPR experiments showed that S (upper panels), S1 (lower left panel) and RBD (lower right panel) bind raloxifene. The sensorgrams (black) were obtained after subtraction of the signal observed on the reference (empty) surface, to show the specific binding signal. For each target protein, the entire sensorgrams (i.e. association and dissociation phases) obtained with three raloxifene concentrations, were globally fitted using the 1:1 Langmuir model. Red lines show the resulting fitting while the corresponding binding parameters are shown in the insets: kon and koff are the association and dissociation rate constants, respectively, while K_D_ is the equilibrium dissociation constants.

High-Performance Computing (HPC) simulation was conducted to generate a profile of raloxifene against SARS-CoV-2 proteins. The simulation was performed with LiGen™ (Ligand Generator) software. Figure 4A reports the docking scores obtained on the selected targets. To further investigate the virus entry machinery, we decided to experimentally validate by Surface Plasmon Resonance (SPR) the binding between raloxifene and the Spike protein (S). We describe for the first time that raloxifene has direct binding affinity for S, its S1 domain and the Receptor Binding Domain (RBD). The affinity was assessed by flowing the compound on the three proteins immobilized on parallel surfaces of the same sensor chip. S, S1 and RBD were immobilized by direct coupling amine chemistry or captured via a Fc-tag. Raloxifene binds the target proteins in a specific and concentration-dependent manner (Fig. 4B). The sensorgrams (black lines) fit well (red lines) with the Langmuir equation, thus allowing to calculate the binding parameters; raloxifene binds S with a dissociation constant (K_D_) ranging from 45.7 to 32.0 µM, depending on immobilization, while K_D_ is 41.7 and 48.5 µM for S1 and RBD, respectively. SPR experiments confirmed a specific and dose-dependent binding of raloxifene to S.

### Raloxifene does not interfere with SARS-CoV-2 entry

**Fig. 5:**
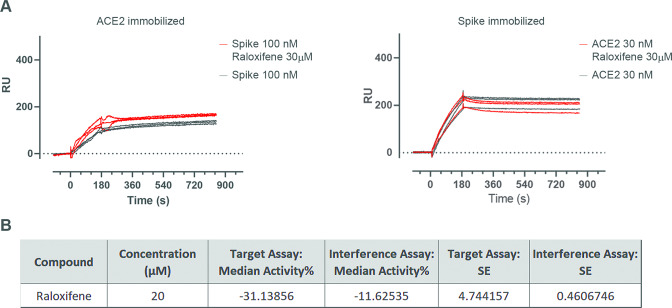
Effect of raloxifene on ACE2-S protein interaction. **A** SPR experiments showed that raloxifene does not interfere with either S binding to immobilized ACE2 (left panel) or with ACE2 binding to immobilized S (right panel). Solutions were injected in triplicate; black lines show the sensorgrams with the proteins alone, while red lines show the sensorgrams with the proteins preincubated with raloxifene. **B** TR-FRET ACE2-SpikeS1 interaction assay showed that raloxifene is not able to interfere with the protein binding.

We used different approaches to identify a possible role of raloxifene in controlling ACE2-S interaction [67, 68]. In the SPR experiments, ACE2 and S were preincubated in the absence or presence of the drug, and then flowed over the corresponding immobilized target proteins (S and ACE2, respectively); no influence of raloxifene in ACE2-S binding was observed (Fig. 5A). Raloxifene was not also able to interfere with ACE2-S1 binding observed in the FRET-based assay (Fig. 5B). Although we cannot completely exclude that raloxifene interferes with the entry mechanisms in other conditions, these data may suggest that other S-mediated processes could be involved in raloxifene-S binding.

### In vitro effect of raloxifene on S-induced ADAM17 mRNA expression

**Fig. 6:**
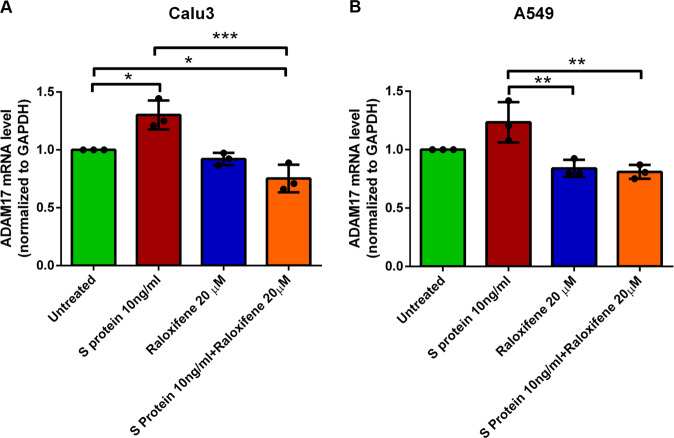
Raloxifene treatment reduces ADAM17 transcriptional levels increased by S exposure. Effect of 48 hours treatment on modulation of ADAM17 mRNA in Calu-3 (**A**) and A549 (**B**) cell line treated with S protein (S 10 ng/ml), raloxifene (Raloxifene 20μΜ) and combined treatments (S 10 ng/ml + Raloxifene 20μΜ). Results are expressed as mean ± SD of 3 independent experiments. *p < 0.05, **p < 0.01, ***P < 0.001.

To test the hypothesis that raloxifene can interfere with S-induced signalling involved in viral replication without affecting S/ACE2 binding, the direct effect of S and raloxifene on ADAM17 in pulmonary cells, such as Calu-3 an A549 cells, was checked. Treatment with recombinant S and raloxifene on ADAM17 modulation was monitored at transcriptional level for 48 h to verify a possible effect of raloxifene alone in unstimulated cells (Fig. 6A, B). Interestingly, in Calu-3 cells (Fig. 6A) in the presence of S we observed an upward trend in ADAM17 mRNA expression levels, significantly inhibited by treatment with raloxifene (− 40%), as compared to S. The modulation of ADAM17 gene expression in A549 cells by treatments confirmed the observed trend. In fact, the treatment with S protein (Fig. 6B) induced an increase of ADAM17 levels which was significantly counteracted by raloxifene.

## Discussion

Raloxifene was proposed as potential candidate for the treatment of COVID-19 due to the in silico predicted possibility to interfere with viral replication and disease progression *via* multiple mechanisms of action, both ER-dependent and independent [43]. Based on these observations, a clinical study was conducted (NCT05172050) in patients with mild to moderate COVID-19 whose results are being published (Nicastri E. et al., 2022 eClinicalMedicine, accepted for publication). This study compared the approved pharmacological dosage of raloxifene 60 mg/day, used to treat and prevent osteoporosis in postmenopausal women, with the dosage of 120 mg/day, also extensively evaluated in clinical trials, to confirm the safety and tolerability of the drug in the indication, and to compare the efficacy of the two doses with the aim of gaining further information on possible ER-dependent or independent mechanisms of action. Previous data from screening campaigns reported the antiviral activity of raloxifene [59]; in this paper we report an in-depth in vitro characterization of the antiviral activity of the drug against the WT and the most common variants of SARS-CoV-2. First of all, we confirmed the anti-CPE of the drug in two relevant experimental systems, Vero E6 monkey kidney cells, commonly used to study coronavirus infection [69–71] and human pulmonary Calu-3 cells, a predictive model of airway epithelial origin [72]. Interestingly, raloxifene was tested against all the most common circulating SARS-CoV-2 VOCs, confirming that the drug retains a high and consistent activity, thus reinforcing the interest on its potential use as antiviral agent in COVID-19. In the two cellular systems here described, raloxifene CC_50_ was set at high micromolar range, far from the low micromolar range of the observed antiviral activity, and the SI was superimposable, ranging from 2 to 7, in line with the expected characteristics for translation into human trials [73, 74]. Among SERMs, raloxifene has a unique benefit/risk profile obtained from its extensive safety database due to its use in cancer patients, in postmenopausal women, and in men [75, 76]. The occurrence of thromboembolic events, even though rare, must be regarded with caution, due to the risk of thromboembolic manifestations in COVID-19 patients, thus a short duration of treatment is recommended and avoidance of treating patients with concomitant risks of thromboembolic events. The potential of SERMs, in particular raloxifene, for the treatment of COVID-19 found a promising confirmation in a recent retrospective study in a population of cancer patients in which protection from SARS-CoV-2 infection and significant reduction in severity and duration of the infection were observed in the subpopulation of patients treated with raloxifene [77]. To strengthen the hypothesis that raloxifene could exert a polypharmacological action in COVID-19 patients, the results of a system biology study strongly suggest the positive influence of the drug during the viral infection due to the modulation of several human genes playing key roles in the biology of the viral infection. The study matched the available information on genes and pathways regulated by raloxifene against a Cytoskape-generated human SARS-CoV-2 interactome network. Data analysis confirmed a putative antiviral activity of raloxifene, highlighting a potential anti-inflammatory action, a vasculo-protective activity through upregulation of eNOS expression (NOS3) [63, 78], and an antiviral activity by modulating key pathways of viral entry and replication, such as ACE2, TMPRSS2 and ADAM17 [66]. Modulation of ER-activated pathways is likely to be only partially responsible for its antiviral activity, since it has been found conserved within the SERM class, but with non-superimposable structure-activity relationship. Several mechanisms have been proposed to support the antiviral action of raloxifene [79, 80]. In agreement with EXSCALATE predictions [43], raloxifene was reported to inhibit SARS-CoV-2 3C-like protease (3 CLpro) viral protein with an IC_50_ value of 5.61 μM [81] that could partially explain the observed in vitro activity. We also checked in this paper its affinity for S and found a low affinity although a specific binding, that could account at least in part for the observed antiviral mechanism. In fact, even if the direct affinity of raloxifene towards S alone is relatively low, we cannot exclude that this binding becomes relevant in case of protein-protein interaction. Surprisingly, and in apparent contradiction with previous studies [81] (https://opendata.ncats.nih.gov/covid19/databrowser), we did not observe a significant inhibition of ACE2-S interaction. Even if we can’t completely rule out the possibility that in other experimental conditions raloxifene can block the viral entry process, our results are perfectly aligned with the observation that the anti-viral activity on Vero E6 and Calu-3 cells occurs only in the post-entry conditions. To evaluate possible alternative effects of raloxifene on S-induced biological activities, we investigated in Calu-3 and A549 cells the direct effect of S on the expression of ADAM17, known for playing a key role in ACE2 shedding, and whose inhibition previously resulted to have a protective effect on SARS-CoV infection [82]. The observed increase of ADAM17 mRNA expression induced by S in both Calu-3 and A549 cells, and its corresponding reduction in presence of raloxifene, represent a first evidence of the drug ability to interfere with S-induced biological activity, downstream the interaction with the membrane ACE2 receptor. ADAM17 modulation may explain the observed in vitro effects but can also suggest a positive in vivo regulation of the anti/pro-inflammatory process balance, tissue regeneration and remodeling [60, 83]. These results confirm that raloxifene may exert a pharmacological action useful to prevent disease progression and exacerbation by direct interaction with viral proteins and/or modulation of ER signaling. One of the main goals of this study was the characterization of raloxifene on the most frequent circulating viral variants because VOCs pose a threat to the ongoing efforts to control the COVID-19 pandemic due to the hypothesis that the infection, originated in bats underwent multiple recombination events during migration through other mammals [7]. Evidence that the new VOCs have acquired the ability to expand species tropism to murines [9] suggests the possibility that these species act as a zoonotic viral reservoir. VOCs diffusion and evolution confirm the need to address multiple mechanisms to inhibit viral replication of the new emerging SARS-CoV-2 variants, and our results, remarkably, highlight the independence of raloxifene activity from individual viral variants. Based on our knowledge, raloxifene is one of the first examples of treatment efficacious against the main circulating SARS-CoV-2 variants, and our results suggest that it could represent a useful additional option to the therapeutic armamentarium for the management of COVID-19 patients in the next years.

## Supplementary information

Supplementary Information

Reproducibility checklist

## Data Availability

All data generated or analysed during this study are included in this published article and in article supplementary material.
