# Supplementary material for: Characterization of raloxifene as a potential pharmacological agent against SARS-CoV-2 and its variants
Source: Cell Death Dis. 2022 May 25;13(5):498. doi: 10.1038/s41419-022-04961-z (PMC9130985; doi:10.1038/s41419-022-04961-z)
Supplement: Supplementary file 1 — Supplementary Information [file 41419_2022_4961_MOESM1_ESM.docx]

**Supplementary Information**

In this section more detailed information on Materials & methods are reported

**Characterization of raloxifene as potential pharmacological agent against SARS-CoV-2 and its variants**

Daniela Iaconis^1*^, Licia Bordi^4*^, Giulia Matusali^4*^, Carmine Talarico^1^, Candida Manelfi^2^, Maria Candida Cesta^2**^, Mara Zippoli^1^, Francesca Caccuri^3^, Antonella Bugatti^3^, Alberto Zani^3^,Federica Filippini^3^, Laura Scorzolini^4^, Marco Gobbi^5^, Marten Beeg^5^, Arianna Piotti^5^, Monica Montopoli^6^, Veronica Cocetta^6^, Silvia Bressan^6^, Enrico Bucci^7^, Arnaldo Caruso^3^, Emanuele Nicastri^4^, Marcello Allegretti^2^ & Andrea Rosario Beccari^1^

^1^Dompé farmaceutici S.p.A., Naples, Italy

^2^Dompé farmaceutici S.p.A., L’Aquila, Italy

^3^Department of Molecular and Translational Medicine, Section of Microbiology and Virology, University of Brescia Medical School, Brescia, Italy.

^4^National Institute for Infectious Diseases Lazzaro Spallanzani-IRCCS, Rome, Italy

^5^Department of Biochemistry and Molecular Pharmacology, Istituto di Ricerche Farmacologiche Mario Negri IRCCS, Milan, Italy

^6^Department of Pharmaceutical and Pharmacological Sciences, University of Padua, Padua, VIMM Veneto Institute Molecular Medicine, Padua, Italy

^7^Sbarro Health Research Organization, Biology Department CFT, Temple University, Philadelphia, PA, USA

**Cells**

African green monkey kidney VeroE6 cell line was obtained from American Type Culture Collection (ATCC, Manassas, VA, USA) and maintained at 37°C in a humidified atmosphere of 5% CO_2_ in Dulbecco's Modified Eagle Medium (DMEM; Gibco, Thermo-Fisher, Waltham, MA, USA). Calu-3 (human, Caucasian, lung, adenocarcinoma) cell line was obtained from ATCC and maintained in Minimum Essential Medium (MEM; Gibco, Thermo-Fisher). Both media were supplemented with 10% fetal bovine serum (FBS; Gibco, Thermo-Fisher)

**Virus**

Genomic data of SARS-CoV-2, belonging to the B.1 lineage, are available at EBI (under study accession number: PRJEB38101^1,2^. Different SARS-CoV-2 variants isolated from COVID-19 patients’ respiratory samples were used. The identity of each variant was verified by metagenomic sequencing.

Below the list of the viral strains used to assess the activity of raloxifene against viral variants:

- Human 2019-nCoV strain 2019-nCoV/Italy-INMI1, clade V (Ref-SKU: 008V-03893, EVAg portal), and isolated in January 2020 from a chinese patient (control infection) (named Wuhan)
- SARS-CoV-2 isolate SARS-CoV-2/Human/ITA/PAVIA10734/2020, clade G, D614G (S) (Ref-SKU: 008V-04005, EVAg portal), named D614G, isolated in Lombardy in February 2020
- SARS-CoV-2 isolate hCoV-19/Italy/LAZ-INMI-82isl/2020, clade GV, A222V, D614G (S) (Ref-SKU: 008V-04048, EVAg portal), named GV and representig the dominant strain circulating in Europe from April to December 2020.
- SARS-CoV-2 variant VOC 202012/01, isolate hCoV-19/Italy/CAM-INMI-118isl/2020, clade GR, Δ69-70, Δ144, N501Y, A570D, D614G, P681H, T716I (S) (Ref-SKU: 008V-04050, EVAg portal), named VOC B.1.1.7 and representing the variant of major concern from UK
- SARS-CoV-2 variant GR/501Y.V3, isolate hCoV-19/Italy/LAZ-INMI-216isl/2021, clade GR, PANGO lineage P.1, K417T, E484K, N501Y (S) (Ref-SKU: 008V-04101, EVAg portal), named VOC P1 representing the variant of major concern from Brazil
- SARS-CoV-2 variant VOC SA/B.1.351, obtained by GHSAG (Public Health England), named B.1.351 and representing the variant of major concern from South Africa
- SARS-CoV-2 variant VOC G Delta /B1.617.2 isolate hCoV-19/Italy/LAZ-INMI-648/2021 (EPI_ISL_2000624) , named VOC B.1.617.2 representing the variant of major concern from India.

All the infection experiments were performed in a biosafety level-3 (BLS-3) laboratory at a multiplicity of infection (MOI) of 0.05.

**Cell viability** **studies of raloxifene**

Cells were seeded into 24-well plates (2.5x10^4^ cells/well) in DMEM supplemented with 10% FBS, and treated with different doses of raloxifene (1.25, 2.5, 5, 10, 15, 20, 25 and 30 μM) at 37°C for 48 h. Cell viability was estimated by measuring the ATP levels using CellTiter-Glo (Promega, Madison, WI, USA).

**Evaluation of antiviral efficacy of raloxifene**

Cells were infected at 37°C for 1 h with the SARS-CoV-2 isolate at a MOI of 0.05. Infection was carryed out in DMEM without FBS. Then, the virus was removed and cells washed with warm phosphate buffered saline (PBS) and cultured with medium containing 2% FBS in the presence or in the absence of raloxifene. The compound was used at the concentration of 1.25, 2.5, 5, 10 and 15 μM and both cells and supernatants were collected for further analysis 48 h post infection (p.i).

**Plaque Assay**

Cells were seeded at a density of 5x10^5^ cells/well in a 12-well plate and incubated at 37°C for 24 h. Supernatants from infected cells were serially diluted in DMEM without FBS and added to the cells. After 1 h incubation, media were removed and cells washed with warm PBS. Then cells were covered with an overlay consisting of DMEM with 0.4% SeaPlaque (Lonza, Basel, Switzerland). The plates were further incubated at 37°C for 48 h. Cells were fixed with 10% formaldehyde at room temperature for 3 h. Formaldehyde was aspirated and the agarose overlay was removed. Cells were then stained with crystal violet (1% CV w/v in a 20% ethanol solution), and viral titer (Plaque Forming Unit, PFU/mL) of SARS-CoV-2 was determined by counting the number of plaques.

**Viral RNA extraction and quantitative real-time RT-PCR (qRT-PCR)**

RNA was extracted from clarified cell culture supernatants (16,000 g x 10 min) and from infected cells using QIAamp Viral RNA Mini Kit and RNeasy Plus mini kit (Qiagen, Hilden, Germany), respectively, according to the manufacturer’s instructions.

RNA was eluted in 30 μl of RNase-free water and stored at -80 °C till use. The qRT-PCR was carried-out following previously described procedures with minor modifications^3^. Briefly, reverse transcription and amplification of the S gene were performed using the one-step QuantiFast Sybr Green RT-PCR mix (Qiagen) as follows: 50^o^C for 10 min, 95^o^C for 5min; 95^o^C for 10 sec, 60^o^C for 30 sec (40 cycles) (primers: RBD-qF1: 5’-CAATGGTTTAACAGGCACAGG-3’ and RBD-qR1: 5’-CTCAAGTGTCTGTGGATCACG-3). A standard curve was generated by determination of copy numbers derived from serial dilutions (10^3^-10^9^ copies) of a pGEM T-easy vector (Promega, Madison, WI, USA) containing the receptor binding domain of the S gene (primers: RBD-F: 5’-GCTGGATCCCCTAATATTACAAACTTGTGCC-3’; RBD-R: 5’-TGCCTCGAGCTCAAGTGTCTGTGGATCAC-3’).

**Western blot analysis**

Western blot was carried-out following previously described procedures with minor modifications^4^. Protein samples (30µg) obtained from lysis in RIPA buffer (Cell Signaling Technology, Danvers, MA, USA) of infected cells were separated on 10% SDS-PAGE and then transferred onto polyvinylidene difluoride (PVDF) membranes (Millipore, Sigma, Burlington, MA, USA). After being blocked with 3% BSA in TBS buffer containing 0.05% Tween 20, the blot was probed with a human serum (1:1000 dilution) containing IgG to the SARS-CoV-2 nucleoprotein (NP) and with mouse anti-human GAPDH monoclonal antibody (G-9: Santa Cruz Biotechnology, Dallas, TX, USA). The antigen-antibody complexes were detected using peroxidase-conjugated goat anti-human or goat anti-mouse IgG (Sigma) and revealed using the enhanced chemiluminescence (ECL) system (Santa Cruz Biotechnology).

**Evaluation of antiviral efficacy of raloxifene on SARS-CoV2 variants**

Vero E6 cells were infected at 37°C for 1h with the SARS-CoV-2 strains indicated in the Virus section at a MOI of 0.05 in 96 well plates. Infection was carryed out in MEM (Sigma) without FBS (Gibco). Then, the virus was removed and cells washed with warm phosphate buffered saline (PBS) and cultured with medium containing 2% FBS in the presence or absence of raloxifene at different doses (0.23, 0.47, 0.94, 1.88, 3.75, 7.5, 15 μM) at 37°C and 5% CO_2_ up to 72h. To determine antiviral efficacy of raloxifene, cell viability and viral induced cytopathic effect (CPE) were mesured in not infected and infected cells treated with serial dilution of the drug, staining the cells with a solution of Crystal Violet (Diapath) and 2% formaldehyde. After 30 min, the fixing solution was removed by washing with tap water, and cell viability was measured by a photometer at 595 nm (Synergy™ HTX Multi-Mode Microplate Reader, Biotek, Winooski, VT, USA).

The percentage of viable cells for each condition was calculated compared to infected-not-treated (set as 0%) and not-infected-not-treated cells (set as 100%). The effect of raloxifene on cell viability was also checked by crystal violet 2% formaldheyde staining in each experiment performed for SARS-CoV-2 variants study.

**System Biology Screening**

We started from the following genes, identified as relevant for the pathogenic effect of SARS-CoV-2 infection by The Host Genetic Initiative (<https://www.covid19hg.org/results/r3/>):: ANKRD32, CDRT4, PSMD13, ERO1L, LZTFL1, XCR1, FYCO1, IFNAR2, CXCR6, CCR9, AP000295.9, AK5. Based on a lookup of previous GWAS results in the GWAS ATLAS database (a database of publicly available GWAS summary statistics), these genes are considered primarily implicated in immunological phenotypes. Then, we looked at the human-SARS-CoV-2 interactome network as published^5^, and extracted all the human genes included in the set. A list combining the two dataset was used as seeding for a BioGrid search by mean of Cytoscape v.3.8.0; the resulting enriched functional network connected those human proteins, known to directly bind SARS-CoV-2 proteins, with the human gene products involved in the host pathology. Subsequently, we screened 8721 Scopus-derived documents, referred to raloxifene, for the presence of at least one of the proteins/genes included in the Cytoscape-generated network; this allowed to isolate 600 papers, which were manually examined and annotated for enriched human gene ontology according to BiNGO v.3.5.0.

**LiGen^TM^ Virtual Screening Protocol**

The geometrical docking procedure implemented in LiGen™, a proprietary software developed by Dompé, was used for the docking simulations. The Pacman Score (PS) estimates a geometric ﬁtting score to evaluate the interaction between a ligand conformation and the pocket, basing on shape and volume information; then the Chemical Score (CS), representing the ligand binding energy, is calculated by using an in-house developed scoring function^6^. Lastly, a minimization algorithm that treats the docket ligand as a rigid body inside the binding site, called the Optimized Chemical Score (CSopt), was evaluated. Furthermore, all computed poses produced during the virtual screening campaigns were rescored using Rescore+. The primary scoring functions plus the scores computed by rescoring were used to generate consensus linear equations through the Enrichment Factor Optimization (EFO) method^7^, as implemented in the VegaZZ suite of programs. The proteins analyzed and the corresponding PDB codes were: 3CL protease, 6LU7; N-protein, 6VYO; NSP3, 6W02; NSP6, De novo model; NSP9, 6W4B; NSP12, 7BV2; NSP13, 6XEZ; NSP14, Homology Model; NSP15, 6W01; NSP16, 6W4H; PL protease, 6W9C; Spike-ACE2, 6M0J; ACE2, 6M17; TMPRSS2, 7MEQ.

**Ligand preparation**

To proceed in the computational study, raloxifene was converted to 3D and prepared with Schrödinger’s LigPrep tool. This process generated multiple states for stereoisomers, tautomers, ring conformations (1 stable ring conformer by default) and protonation states. In particular, another Schrödinger package, Epik, was used to assign tautomers and protonation states that would be dominant at a selected pH range (pH=7±1). Ambiguous chiral centers were enumerated, allowing a maximum of 32 isomers to be produced from each input structure. Then, an energy minimization was performed with the OPLS3 force.

**Surface Plasmon Resonance (SPR) analysis**

All analyses were carried out with a ProteOn XPR36 Protein Interaction Array system (Bio-Rad Laboratories, Hercules, CA) SPR apparatus that immobilizes different ligands in parallel surfaces on the same AL1 alginate sensor chip (Xantec GmbH). Ee investigated if raloxifene, flowing in the microfluidic channels, bind the target proteins (Spike protein, its S1 domain and RBD) immobilized on the sensor chip surface. The specificity of the binding is established by comparison with a reference surface prepared in the same manner but without the target protein. Spike protein (Euprotein) was immobilized either by direct amine coupling or captured via its Fc-tag using a previously immobilized anti-Fc antibody (Merck Life Science S.r.l). S1 domain and RBD (SinoBiological), both Fc-tagged, were also captured via the anti-Fc antibody. In particular, FcS, FcS1 or FcRBD were flowed on the anti-Fc antibody at 30 µg/mL in 10mM phosphate buffer containing 150 mM NaCl and 0.005% Tween 20 (PBST, pH 7.4), also used as running buffer. The level of immobilization ranged from 1600-2500 Resonance Units (RU). The flow channels were then rotated 90° so that raloxifene could be flowed in parallel on all the immobilized ligands and the reference surface. All consecutive SPR assays were run at a rate of 30μL/min at 25°C. The binding was evaluated injecting for 180 secs the compound at 3, 10 and 30 μM, in PBST pH 7.4 with 0.33% DMSO, allowing dissociation times in between, with no regeneration steps. The sensorgrams (time course of the SPR signal in RU) were normalized to a baseline of 0. The association and dissociation rate constants (kon and koff) and the equilibrium dissociation constant (K_D_) were obtained from globally fitting the Langmuir model, implemented in ProteOn analysis software, to entire sensorgrams (association and dissociation phases) obtained by injecting different raloxifene concentrations. To evaluate the ability of raloxifene to inhibit the ACE2-S protein interaction, we used an indirect experimental design. 30 nM ACE2 or 100nM of Spike protein were preincubated in the absence or presence of 30μM of raloxifene, for 60 min at room temperature, and then flowed over the corresponding target protein (Spike protein or ACE2, respectively) immobilized by amine coupling (immobilization level 9100 RU and 8600 RU for Spike protein or ACE2, respectively). The running buffer was PBST with 0.33% DMSO.

**ACE2-SpikeS1 interaction assay**

This assay is a TR-FRET-based protein-protein interaction assay (AXXAM SpA proprietary assay). This assay is a TR-FRET-based protein-protein interaction assay in which HIS tagged SpikeS1 interaction with FLAG tagged ACE2 was mesured. The complex brings donor labeled anti HIS antibody (Mab Anti-6HIS Tb cryptate Gold) and acceptor labeled anti FLAG antibody (Mab Anti FLAG M2XL665) in close proximity. Excitation of the donor at 320 nm results in energy transfer to the acceptor and emission at a higher wavelength after a time delay. Thus, the interaction between ACE2 protein and SpikeS1 protein is measured as increase of fluorescent signal and the inhibitory activity of a drug as a decrease of the fluorescence signal. ACE2-Fc competing peptide was designed as a positive control for inhibition, while an interference assay was run to test aspecific effect on fluorocrome in which HIS tagged SpikeS1 and FLAG tagged ACE2 were replaced by an unrelated peptide with a FLAG tag and a HIS tag. Reaction buffer 1X: HEPES-Na pH 8.0 10 mM, NaCl 150 mM, Tween 20 0.05 %, faf-BSA 0.005 %. Fluorescent signal was measured on the Pherastar FSX (BMG Labtech).

**Evaluation of activity of raloxifene on ADAM-17 mRNA expression-RNA extraction and qRT-PCR**

Cells were seeded in 6 well plates and after incubation, were treated according to the experimental protocol with Spike protein (10ng/ml) and raloxifene (20μΜ). Total RNA was extracted from cell lines using RNeasy® Plus Mini Kit (Qiagen). cDNA was then made using GenePro thermal cycler (Bioer). RT-PCR analysis to target mRNAs was performed on QuantStudio5 Applied Biosystem (Thermo Fisher Scientific) using Itaq™ Universal SYBR (Bio-Rad) gene expressions assays. Primers for GAPDH (forward primer AATCCCATCACCATCTTCCA; reverse primer TGGACTCCACGACGTACTCA) and ADAM17 (forward primer GTGGATGGTAAAAACGAAAGCG; reverse primer GGCTAGAACCCTAGAGTCAGG) were used. Samples were assayed in runs which were composed of 3 stages: hold stage at 95°C for 20 min, PCR stage at 60°C for 25 minutes and melt curve stage 95°C for 1 minute, 60°C for 20 min, and 95°C for 1 min again. Gene expressions were normalized by GAPDH per sample and then compared to the control sample untreated to determine relative expression values by the 2^-ΔΔCt^ method.

Statistical Analysis: data were analyzed for statistical significance using the 1-way ANOVA. Differences were considered significant when p < 0.05. Statistical tests were performed using GraphPad Prism 8.

**Data analysis**

The half-cytotoxic concentration (CC_50_) and the half-maximal inhibitory concentration (IC_50_) for raloxifene were calculated from concentration-effect-curves after non-linear regression analysis using GraphPad Prism8. The selectivity index (SI) for raloxifene was calculated as the ratio of CC_50_ over IC_50_^8^.

**Statistical analysis**

Data for the *in vitro* experiments performed were analyzed for statistical significance using the 1-way ANOVA, and the Bonferroni post-test was used to compare data. Differences were considered significant when *p* < 0.05. Statistical tests were performed using GraphPad Prism 8.

**References**

1. Caruso A, Caccuri F, Bugatti A, Zani A, Vanoni M, Bonfanti P*, et al.* Methotrexate inhibits SARS-CoV-2 virus replication "in vitro". *Journal of medical virology* 2021, **93**(3)**:** 1780-1785.

2. Caccuri F, Zani A, Messali S, Giovanetti M, Bugatti A, Campisi G*, et al.* A persistently replicating SARS-CoV-2 variant derived from an asymptomatic individual. *Journal of translational medicine* 2020, **18**(1)**:** 362.

3. Wang M, Cao R, Zhang L, Yang X, Liu J, Xu M*, et al.* Remdesivir and chloroquine effectively inhibit the recently emerged novel coronavirus (2019-nCoV) in vitro. *Cell Res* 2020, **30**(3)**:** 269-271.

4. Caccuri F, Bugatti A, Meini A, Bonfanti C, Motta M, Savare L*, et al.* Temporal viral loads in respiratory and gastrointestinal tract and serum antibody responses during SARS-CoV-2 infection in an Italian pediatric cohort. *Clin Immunol* 2021, **225:** 108695.

5. Gordon DE, Jang GM, Bouhaddou M, Xu J, Obernier K, White KM*, et al.* A SARS-CoV-2 protein interaction map reveals targets for drug repurposing. *Nature* 2020, **583**(7816)**:** 459-468.

6. Beccari AR, Cavazzoni C, Beato C, Costantino G. LiGen: a high performance workflow for chemistry driven de novo design. *J Chem Inf Model* 2013, **53**(6)**:** 1518-1527.

7. Mazzolari A, Vistoli G, Testa B, Pedretti A. Prediction of the Formation of Reactive Metabolites by A Novel Classifier Approach Based on Enrichment Factor Optimization (EFO) as Implemented in the VEGA Program. *Molecules* 2018, **23**(11).

8. Yuan S, Wang R, Chan JF, Zhang AJ, Cheng T, Chik KK*, et al.* Metallodrug ranitidine bismuth citrate suppresses SARS-CoV-2 replication and relieves virus-associated pneumonia in Syrian hamsters. *Nat Microbiol* 2020, **5**(11)**:** 1439-1448.

**Supplementary Figures**


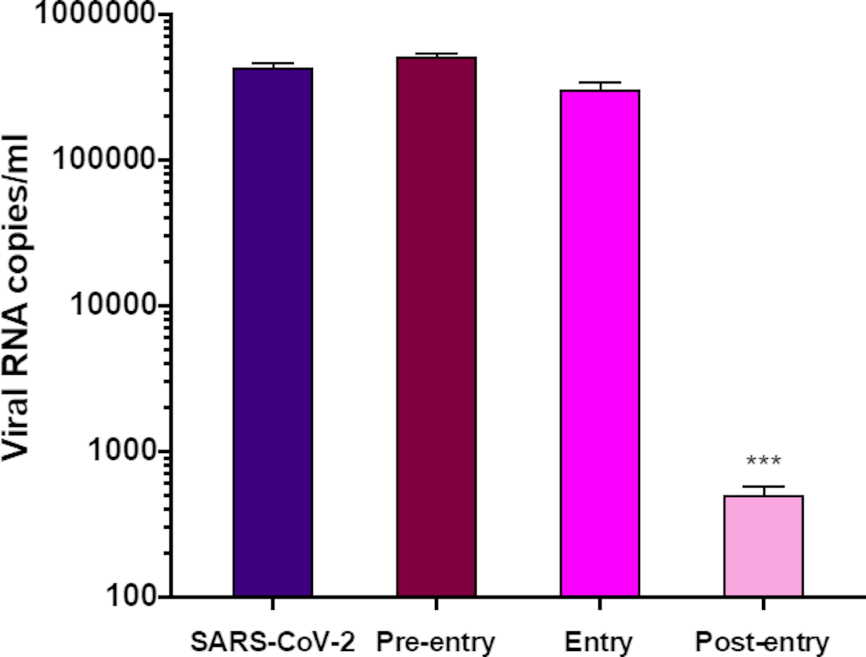


**Fig. S1 Antiviral activity of raloxifene against SARS-CoV-2.** For “Pre-entry” treatment, Vero E6 cells were pre-treated with 15µM of raloxifene for 16hrs, and then the cells were washed and virus was added to allow attachment for 1h. Afterwards, the virus was removed, the cells were washed and then cultured with medium alone until the end of the experiment. For “Entry” treatment, 15µM of raloxifene were added to the cells for 1h during viral attachment, the virus-drug mixture was replaced with fresh culture medium and maintained till the end of the experiment. For “post-entry” treatment, 15µM of raloxifene were added at 1h p.i., and maintained until the end of the experiment. For all the experimental groups, VeroE6 cells were infected with SARS-CoV-2 at a MOI of 0.05, and virus yield in the infected cell supernatants was quantified by qRT-PCR. All the experiments were performed at least in three independent replicates. Data are presented as the mean + standard error of the mean ***, *P* < 0.001.


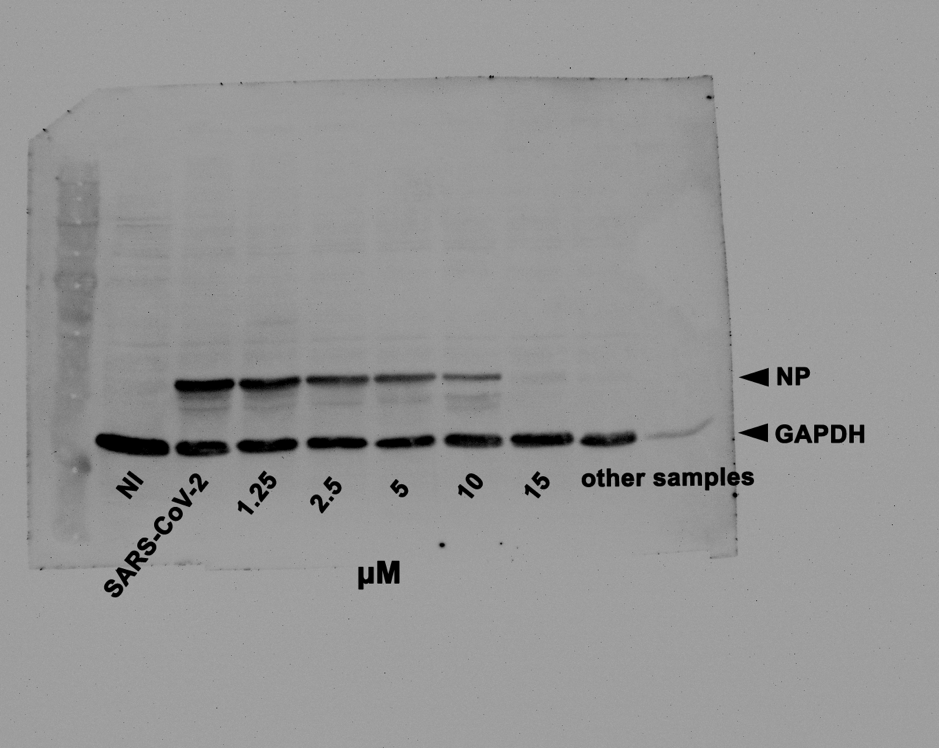


**Fig. S2. Full and uncropped western blot shown in Figure 1F.**

**
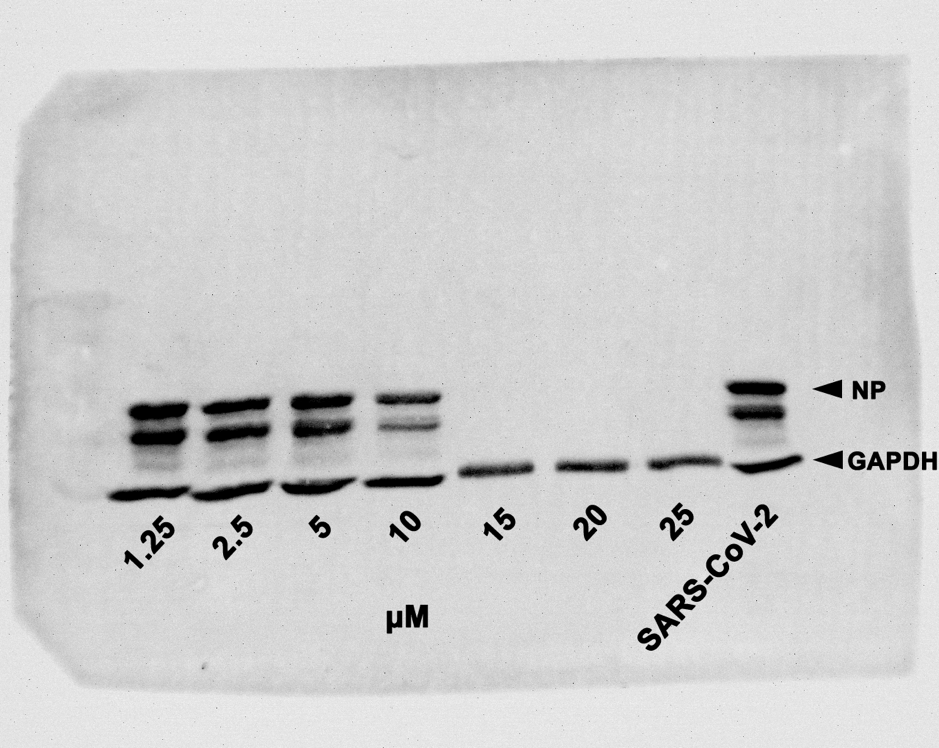
**

**Fig. S3. Full and uncropped western blot shown in Figure 2E.**


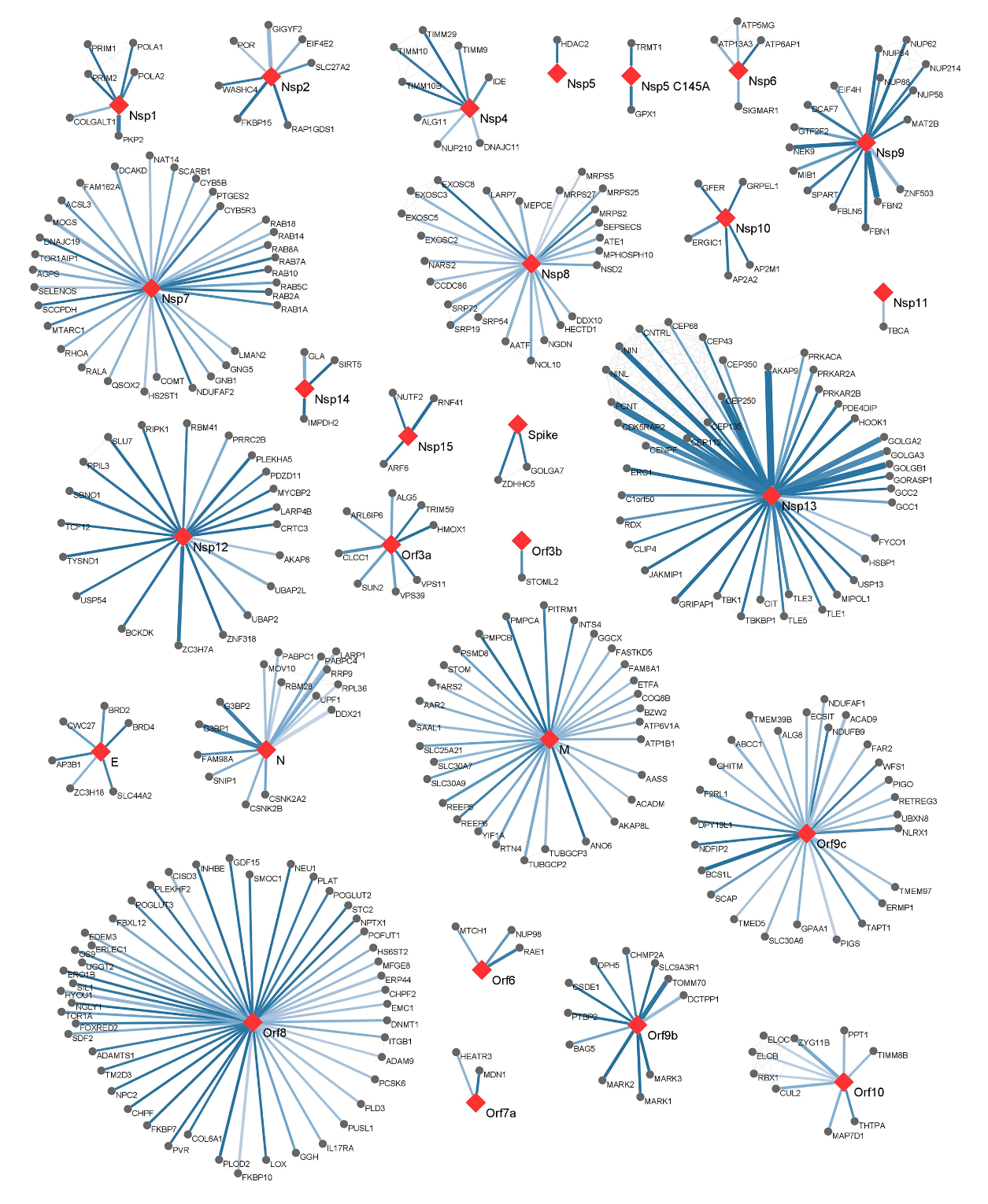


**Fig. S4 SARS-CoV-2 protein-protein interaction network.** Viral proteins are reported as red diamonds, human proteins as grey circles (freely taken from Gordon, D.E., Jang, G.M., Bouhaddou, M. et al. A SARS-CoV-2 protein interaction map reveals targets for drug repurposing. Nature 2020;583:459–468).
